# Screening of patients with augmented renal clearance in ICU: taking into account the CKD-EPI equation, the age, and the cause of admission

**DOI:** 10.1186/s13613-015-0090-8

**Published:** 2015-12-14

**Authors:** Stéphanie Ruiz, Vincent Minville, Karim Asehnoune, Marie Virtos, Bernard Georges, Olivier Fourcade, Jean-Marie Conil

**Affiliations:** Department of Anesthesia and Intensive Care, University Hospital of Toulouse, University Toulouse III Paul Sabatier, Toulouse, France; Service d’Anesthésie Réanimation, CHU de Nantes, 1 place Alexis Ricordeau, 44093 Nantes Cedex 1, France

**Keywords:** Critically ill patients, Measured creatinine clearance, GFR estimations, Screening ARC

## Abstract

**Background:**

In ICU patients with normal serum creatinine (SCr), a state of increased renal drug excretion has been described (creatinine clearance ≥130 ml/min/1.73 m^2^), and named augmented renal clearance (ARC). In ICU patients, the accuracy of GFR estimates is insufficient. However, in clinical practice, the physician has not at one’s disposal patient measured creatinine clearance (CrCl) when prescribing. The primary objective of this study was to assess the accuracy of 4 formulas to estimate GFR (Cockcroft-Gault (CG), Robert, sMDRD, and CKD-EPI formulas) with other covariates to detect ARC in ICU patients.

**Methods:**

We enroled 360 consecutive ICU patients with normal SCr in this prospective observational study conducted in a primary teaching hospital. Comparisons between CrCl values and 4 estimated GFR (eGFR) formulas were estimated.

**Results:**

In these 360 patients, ARC was observed in 33 % of patients most of them trauma. Individual predictive values of equations were poor and the phenomenon increased in ARC subgroup. CG and CKD-EPI were more accurate to detect an ARC. Multivariable analysis showed that the best-fitting model included 3 factors independently correlated to ARC: trauma patients, cut-off values of age ≤58 years, and CKD-EPI more than 108 ml/min/1.73 m^2^.

**Conclusions:**

In ICU patients with normal SCr, eGFR formulas are imprecise in assessing CrCl. If measured CrCl must be ideally used to detect modifications of the renal function, in clinical practice, age, reason for admission, and CKD-EPI could be used as screening tool to identify ARC.

## Background

The glomerular filtration rate (GFR) can affect the pharmacokinetic/pharmacodynamic profile of drugs eliminated by the kidney. The dosages and schedules for the administration of these drugs are traditionally adjusted in patients with a diminished GFR in order to achieve effective plasma levels and to limit drug-induced toxicity. Direct measurement of the GFR with exogenous substances such as inulin is the gold standard for the assessment of renal function, but is not routinely performed in the intensive care units for practical reasons. Instead, one could measure the CrCl from a 24 or 8-h urine collection. However, in clinical practice, the GFR is most commonly estimated (eGFR) from the serum creatinine (SCr), using various formulas including Cockcroft-Gault, Roberts, Modification of Diet in Renal Disease (MDRD), and the 2011 Chronic Kidney Disease Epidemiology Collaboration (CKD-EPI) [1–6].

While critically ill patients can have a decreased GFR with impaired elimination of renally excreted drugs, a state of increased renal drug excretion has also been described and named “augmented renal clearance” (ARC). This state characterized by a creatinine clearance >130 ml/min/1.73 m^2^ has a reported incidence of 30–85 %, depending on the population studied and the cut-off values used for its definition [7–9]. Even though ARC is common in critically ill patients, a dose escalation for those patients is infrequently reported in clinical practice [1, 10–14]. This is probably because a normal SCr in critically ill patients which is not a sensitive indicator of renal dysfunction may induce an underestimation of the actual GFR, meaning that some ICU patients do not achieve adequate plasma levels of their antimicrobial drugs [15–21].

The primary objective of this study was to assess the accuracy of 4 commonly used formulas to estimate GFR with other covariates, to detect “augmented renal clearance” in ICU patients with normal serum creatinine concentrations.

## Methods

### Patients

This observational study was conducted in the ICU at Rangueil Hospital, a primary teaching hospital of the University of Toulouse (France) according to the declaration of Helsinki (approval by Ethical Research Committee of University Toulouse Hospital). Since the CrCl in our ICU is measured routinely at least once a week, the need for informed consent was waived.

All consecutive critically ill patients, older than 16 years, hemodynamically stable, with an arterial catheter, a urinary bladder catheter, and a stable SCr (in the normal range of 40–120 μmol/l; with less than 25 % variation between the 4th and the 10th day after admission) were included. Patients were divided into two groups, according to the diagnosis on admission: polytrauma (PT) and non-polytrauma (NPT) with the latter divided into surgical (SURG) and medical (MED) patients.

Patients were excluded from the study if they were hemodynamically unstable and needed a high dose of catecholamines (norepinephrine >1 mg/h); were recovering from or developing acute kidney injury (AKI); received histamine-2-receptor antagonist due to its interference with tubular creatinine secretion or if they had a medical history of diabetes, chronic liver disease, cirrhosis, or ongoing liver dysfunction with hepatitis [22, 23]. We excluded patients with the history of diabetes and liver disease, because glucose, ketoacids, and bilirubin are common interfering agents which lead to the overestimation of serum creatinine by Jaffe methods [24]. Patients treated with diuretics were also excluded.

Baseline characteristics for patients were recorded at enrolment in the study, and the SAPS II and SOFA scores were taken from the time of ICU admission.

### Data collection

Clinical and biological data were collected between the 4th and the 10th day after admission, as soon as the patient met the inclusion criteria. Urines were sampled over 24 h for measuring urinary creatinine concentration, and SCr was measured during that same period (modified kinetic Jaffe colorimetric reaction). Measured CrCl was then calculated using the standard formula: CrCl = (UCr × V)/SCr, where UCr (urine creatinine concentration) and SCr were expressed in µmol/l and V corresponded to the urinary flow rate (diuresis) in ml/min. At the same time, the CrCl was calculated using different formulas, i.e., the Cockcroft formula ${\text{CrCl}}\; = \;\frac{{(140\; - \;{\text{age}})\; \times \;{\text{weight}}}}{{0.8\; \times \;{\text{SCr}}}}$ for men, with age in years and weight in kg [2]. A correcting factor of 0.85 was used for women. We adjusted the Cockcroft formula on body surface area (BSA) of 1.73 m^2^. The BSA was calculated as BSA (m^2^) = [weight (kg) × height (cm)/3600]^1/2^ [25]. We used weight at inclusion to calculate the Cockcroft formula and BSA. The formula proposed by Robert et al. uses the ideal body weight and serum creatinine concentration corrected to 85 µmol/l when the actual value is lower than 85 µmol/l [3]. Ideal body weight was determined as 50 kg for men and 45.5 kg for women, plus 2.3 kg for each inch >5 feet [26].

As per convention, CrCl values were normalized to a body surface area (BSA).

The following simplified Modification of Diet in Renal Disease equation (sMDRD) was used: sMDRD = 186.3 × SCr^−1.154^ × Age^−0.203^ × [1.212 if black], where SCr was expressed in mg/dl [5]. At the same time, we also calculated CrCl according to the CKD-EPI equation, taking into account SCr, gender and ethnicity as follows [6]:

Female

$${\text{Serum creatinine}}\;\upmu {\text{mol}}/{\text{l }}\left( {{\text{mg}}/{\text{dl}}} \right)\; \le \; 6 2 { }\left( { \le 0. 7} \right){:}{\text{ GFR }} = { 144 } \times \, \left( {{\text{SCr}}/0. 7} \right)^{ - 0. 3 2 9} \times \, \left( {0. 9 9 3} \right)^{\text{age}}$$

$${\text{Serum creatinine}}\;\upmu {\text{mol}}/{\text{l }}\left( {{\text{mg}}/{\text{dl}}} \right) \; > \; 6 2 { }\left( { > 0. 7} \right){:}{\text{ GFR }} = { 144 } \times \, \left( {{\text{SCr}}/0. 7} \right)^{ - 1. 20 9} \times \, \left( {0. 9 9 3} \right)^{\text{age}}$$

Male

$${\text{Serum creatinine }}\upmu {\text{mol}}/{\text{l }}\left( {{\text{mg}}/{\text{dl}}} \right) \le 80 \, \left( { \le 0. 9} \right){:}{\text{ GFR }} = { 141 } \times \, \left( {{\text{SCr}}/0. 9} \right)^{ - 0. 4 1 1} \times \, \left( {0. 9 9 3} \right)^{\text{age}}$$

$${\text{Serum creatinine}}\;\upmu {\text{mol}}/{\text{l }}\left( {{\text{mg}}/{\text{dl}}} \right) > 80 \, \left( { > 0. 9} \right){:}{\text{ GFR }} = { 141 } \times \, \left( {{\text{SCr}}/0. 9} \right)^{ - 1. 20 9} \times \, \left( {0. 9 9 3} \right)^{\text{age}}$$

A CrCl ≥ 130 ml/min/1.73 m^2^ was used to define ARC [10, 14].

### Statistical analysis

Data are presented as mean ± standard deviation or ratio. Differences between groups were calculated using parametric and non-parametric tests as appropriate.

The agreement between the individual eGFRs by the CG, Robert, sMDRD, and CKD-EPI formulas and the measured CrCl was analyzed by residual plots according to the method of Bland and Altman [27].

The bias and the precision of the different formulas compared with the measured CrCl were evaluated according to Sheiner et Beal by the following equations [28]:

$${\text{Bias}}\; = \;\frac{1}{N}\sum\limits_{i = 1}^{N} {{\text{CrC}}_{\text{estimated}} \; - \;{\text{CrCl}}} \;\;\;\;{\text{Precision }}\; = \;\sqrt {\frac{1}{N}\sum\limits_{i = 1}^{N} {\left( {{\text{CrCl}}_{\text{estimated}} - {\text{CrCl}}} \right)}^{2} }$$

The results are expressed as a percentage of the mean measured CrCl.

The diagnostic accuracy of the 4 used formulas to estimate GFR and other significant variables in predicting ARC was assessed by measuring the area under the receiver operating characteristic (ROC) curves. Each measure was treated as an independent event. The areas under the ROC curves of the eGFRs were compared by the Wilcoxon rank test. The best threshold with their corresponding likelihood ratios (negative and positive) was defined by Youden’s index.

For each significant variable, the “gray zone” was determined using a two-step procedure as described by Cannesson [29]. The first step consisted of the determination of the best threshold for each parameter. The second step was conducted to determine a range of values for which formal conclusions could not be obtained. We defined inconclusive responses for values presenting with either sensitivity lower than 90 % or specificity lower than 90 % (diagnosis tolerance of 10 %). The gray zone was then defined as the values of the parameters that did not allow having 10 % of diagnosis tolerance. Nevertheless, if the characteristics of the study population produce a 95 % CI of the best thresholds larger than the inconclusive zone, the values obtained during the first step were retained as gray zone.

A logistic regression was performed to determine if polytrauma or any continuous variable or their cut-off with a *p* value of less than 0.20 in the univariate analysis was independently able to predict the presence of ARC. The odds ratio (OR) and 95 % confidence intervals (CI) were calculated. Goodness of fit of the model was assessed using the Hosmer–Lemeshow test [30].

Statistical analysis was performed using Medcalc (MedCalc Software, Ostend, Belgium). A *p* value ≤0.05 was considered as statistically significant.

## Results

### Baselines characteristics of study subjects

**Fig. 1:**
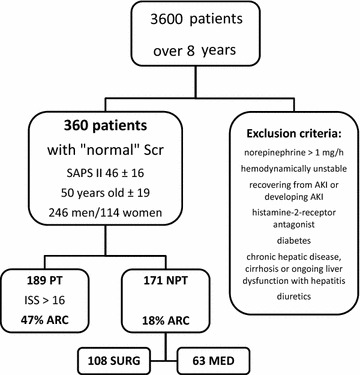
Flow chart showing the process of recruitment

Three hundred and sixty eligible patients completed the study (Fig. 1). The interval from ICU admission to inclusion was 9 ± 5 days; 270 (75 %) of the patients were mechanically ventilated. Demographic and laboratory data are shown in Table 1, including a mean SCr of 72 ± 22 µmol/l.

**Table 1:** Demographic and laboratory data

	Total	Patients with ARC (*n* = 120)	Patients without ARC (*n* = 240)	*p* ^#^
Age (years), mean ± SD	50 ± 19	39 ± 16	55 ± 18.7	<0.0001*
BMI (kg/m^2)^, mean ± SD	25 ± 4.6	24.65 ± 3.85	25.18 ± 4.94	0.3005
BSA, mean ± SD	1.86 ± 0.22	1.89 ± 0.19	1.86 ± 0.22	0.1291
SAPS II, mean ± SD	46 ± 16	43 ± 15	48 ± 16	0.003*
SOFA, mean ± SD	4.3 ± 1.9	4.1 ± 1.6	4.4 ± 2	0.6373
Diagnosis				
PT/NPT	189/171	89/31	100/140	<0.0001*
Sex (F/M)	114/246	30/90	84/156	0.0558
Serum creatinine (µmol/l), mean ± SD	72.14 ± 22.4	63.5 ± 17	76.5 ± 23.6	<0.0001*
Urine (ml/d)	2571 ± 1178	2878 ± 1353	2363 ± 996	0.0018*
Urinary creatinine excretion (mg/d/1.73 m^2^)	1239.7 ± 686.7	1812 ± 758	953.4 ± 419	<0.0001*
Measured CrCl (ml/min/1.73 m^2^), mean ± SD	110.75 ± 56.8	173.4 ± 44.3	79.4 ± 30.4	<0.0001*
CG formula (ml/min/1.73 m^2^), mean ± SD	114.4 ± 41.5	137.6 ± 34.4	98.2 ± 38.5	<0.0001*
Robert formula (ml/min/1.73 m^2^), mean ± SD	79.7 ± 25.9	94.8 ± 24	72.2 ± 23.3	<0.0001*
sMDRD equation (ml/min/1.73 m^2^), mean ± SD	112 ± 40.9	132.5 ± 36.9	101.9 ± 39	<0.0001*
CKD-EPI equation (ml/min/1.73 m^2^), mean ± SD	98.9 ± 25.8	115.4 ± 18.9	90.7 ± 24.9	<0.0001*

*BMI* body mass index, *BSA* body surface area (m^2^), *PT* polytrauma, *NPT* non-polytrauma, *p* ^#^ comparison between patients with and without ARC, * statistically significant

Estimated GFRs based on the various formulas and measured CrCl for patients without (A) and with ARC (B) are presented in Table 2.

**Table 2:** Glomerular filtration rate in ml/min/1.73 m^2^ based on measured creatinine clearance (CrCl), and estimated by the Cockcroft and Gault, Robert, sMDRD, and CKD-EPI formulas in patients without (A) and patients with ARC (B)

(A) Measures of GFR in patients without ARC (*n* = 240) ml/min/1.73 m^2^	Measured CrCl	CG formula	Robert formula	sMDRD equation	CKD-EPI equation
Mean ± SD	79.4 ± 30.4	98.3 ± 38.5	72.2 ± 23.3	101.9 ± 39	90.7 ± 24.9
Coefficient of variation (%)	38.2	39.1	32.3	38.4	27.5
Bias	–	18.8	−7.3	22.5	11.3
Precision	–	31.7	25.1	34.6	25.3

(B) Measures of GFR in patients without ARC (*n* = 120) ml/min/1.73 m^2^	Measured CrCl	CG formula	Robert formula	sMDRD equation	CKD-EPI equation
Mean ± SD	173.4 ± 44.3	137.6 ± 34.4	94.8 ± 24	132.5 ± 36.9	115.4 ± 18.9
Coefficient of variation (%)	25.5	25	25.5	27.9	16.3
Bias	–	−35.7	−78.6	−40.9	−57.9
Precision	–	47.	78.6	51.9	58.3

The incidence of ARC was 120 in the 360 patients (33 %), and the diagnosis of polytrauma was significantly more common among patient with ARC (89/120, 74 %) compared with the non-ARC group (100/240, 41 %, *p* < 0.0001). Patients with ARC were younger: 39 ± 16 years *vs.* 55 ± 19 years in non-ARC patients (*p* < 0.0001). Estimated GFRs were different between patients presenting ARC and the others. Glomerular hypofiltration (CrCl < 60 ml/min/1.73 m^2^) was observed in 21.4 % of the cases. 31 patients were classified as stage III CKD by CKD-EPI equation (eGFR between 30 and 59 ml/min/1.73 m^2^), but 5 of them presented CrCl ≥ 60 ml/min/1.73 m^2^. When stratifying patients based on CrCl > 130 ml/min/1.73 m^2^, CrCl between 60 and 130 ml/min/1.73 m^2^ and CrCl < 60 ml/min/1.73 m^2^, urine creatinine excretion decreases significantly with CrCl (1812.3 ± 757.6 mg/d/1.73 m^2^, 1116.7 ± 388.5 mg/d/1.73 m^2^, and 607.9 ± 229.6 mg/d/1.73 m^2^, respectively). The same occurred with urine output: 2878 ± 1353 ml/d in group with CrCl > 130 ml/min/1.73 m^2^, 2441 ± 1047 in group between 60 and 130 ml/min/1.73 m^2^, and 2216 ± 887 in group with CrCl < 60 ml/min/1.73 m^2^.

### Formulas’ accuracy to estimate CrCl in ICU patients

**Fig. 2:**
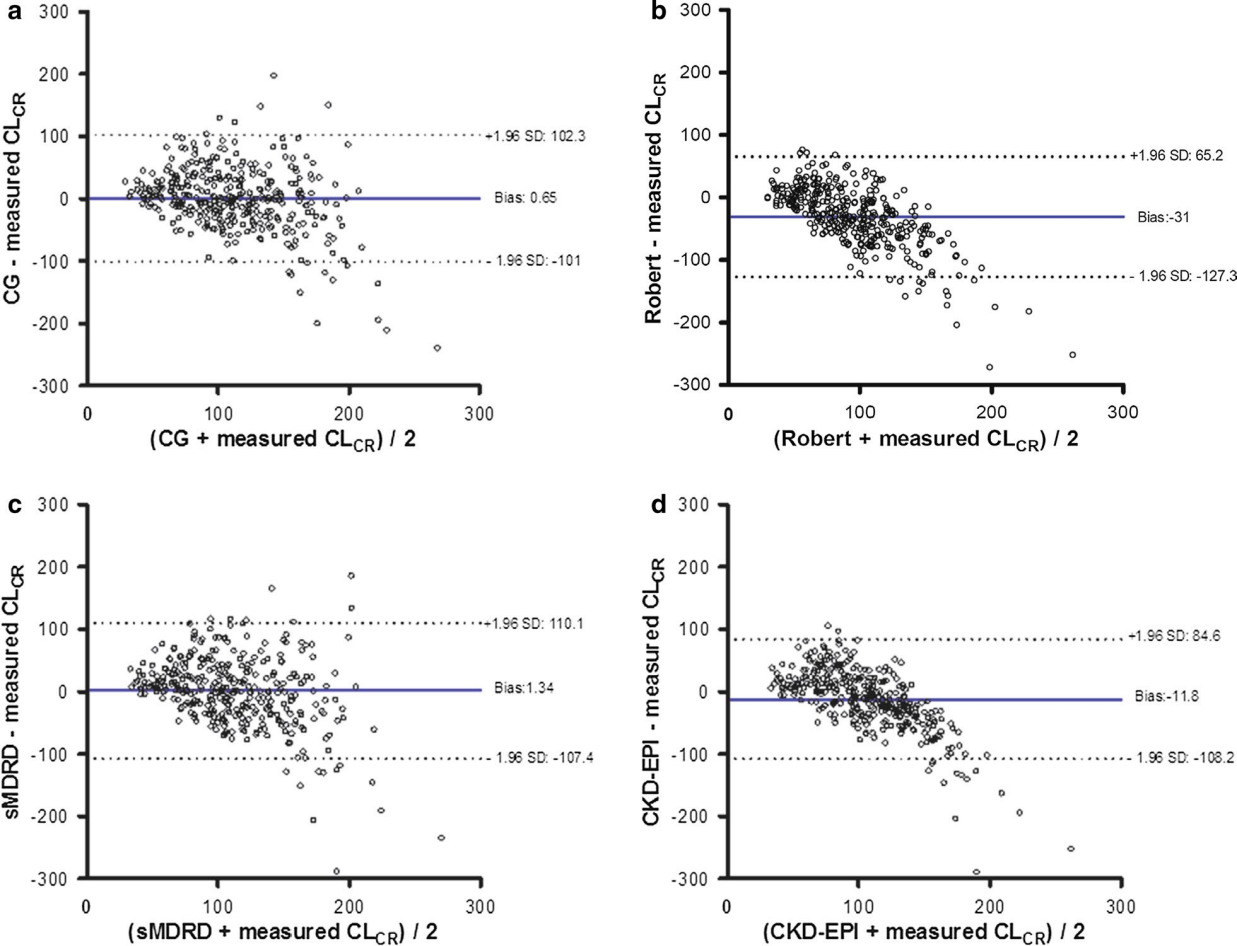
Measures of agreement (Bland and Altman method) between the eGFR by the Cockcroft and Gault (**a**), Robert (**b**), sMDRD (**c**) and CKD-EPI (**d**) formulas and measured creatinine clearance

Estimated GFRs by the 4 equations in patients with and without ARC were significantly different when compared to measured CrCl. Bland and Altman plots are presented in Fig. 2. The different formulas tended to overestimate the CrCl for low eGFR values and to underestimate the CrCl for normal and high eGFRs.

In ARC sub group, each formula underestimated CrCl (Table 2b). For all four formulas, a larger bias and a lower precision were observed in ARC group (Table 2).

### Tools to screen ARC

Age, SAPS II, serum creatinine, and estimated GFRs based on the 4 formulas were different in patients with ARC. The area under the ROC curve of these covariates is presented in Table 3.

**Table 3:** (A) Accuracy of age and SAPS II to detect ARC. (B) Accuracy of SCr, Cockcroft and Gault, Robert, sMDRD, and CKD-EPI formulas to detect ARC

(A)	AUC	IC 95 % AUC	Cut-off	Gray zone	Sensitivity	Specificity	PPV	NPV
Age (years)	0.74	0.69–0.79	≤58	25-60	89.2	52	48	90.5
SAPS II	0.59	0.54–0.64	<54	24-62	76	38	37	76

(B) Formula eGFR	AUC	IC 95 % AUC	Cut-Off	Gray zone	Sensitivity	Specificity	PPV	NPV
SCr	0.66	0.61–0.71	≤71	45.75–88.5	73.33	53.33	44	80
CG	0.79	0.74–0.83	>107.50	95.5–148.6	83.33	62.92	53	88
CKD-EPI	0.79	0.75–0.83	>108.11	90–122.8	75.00	74.58	60	86
Robert	0.75	0.70–0.79	>83.19	63.3–104.7	67.50	70.83	54	81
sMDRD	0.73	0.68–0.78	>107.63	88–150.3	76.67	61.67	50	84

*AUC* area under the curve, *Gray zone* based on Cannesson method, *PPV* positive predictive value (%), *NPV* negative predictive value (%), for SCr cut-off and gray zone are expressed in µmol/l, for eGFRs cut-off and gray zone are expressed in ml/min/1.73 m²

**Fig. 3:**
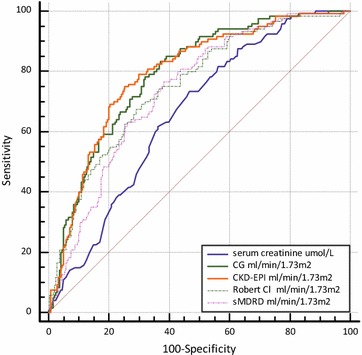
Comparison of ROC curves of serum creatinine and of the 4 estimated GFR formulas to detect ARC. CG and CKD-EPI accurately detect ARC in ICU patients with cut-off values more than 107.5 for CG and 108.11 ml/min/1.73 m^2^ for CKD-EPI

For the screening of ARC, the area under the ROC curve was 0.79 [95 % CI: 0.74–0.83] for the CG formula, 0.75 [95 % CI: 0.7–0.79] for the Robert formula, 0.73 [95 % CI: 0.69–.78] for the sMDRD equation, and 0.79 [95 % CI: 0.75–0.83] for the CKD-EPI equation (Table 3; Fig. 3).

As shown in Table 3, only CG and CKD-EPI formulas presented AUCs above 0.75 to detect an ARC with cut-off values of 107.5 for CG (sensitivity of 83 % and specificity of 63 %) and 108.11 ml/min/1.73 m^2^ for CKD-EPI (sensitivity of 75 % and specificity of 75 %). Considering the gray zones, the limits in which ARC classification could not be reliably predicted were 90–122.8 ml/min/1.73 m^2^ for CKD-EPI equation and 95.5–148.6 ml/min/1.73 m^2^ for CG formula.

Comparison of ROC curves of the 4 estimated formulas show no difference between CG and CKD-EPI AUCs (Fig. 3), but revealed a significant difference between AUC of serum creatinine and CKD-EPI, CG or sMDRD (*p* < 0.0001), and Robert formula (*p* = 0.0243).

We tested several logistic regression models with significant covariates, in particular, the cut-off values of ARC detection by CG and CKD-EPI (Table 4).

**Table 4:** Logistic regression for measured creatinine clearance greater than 130 ml/min/1.73 m^2^

ARC: measured CL_CR_ > 130 ml/min/1.73 m^2^	*p*	Odd ratio [IC 95 %]
(A) Taking into account CG equation (Goodness of fit was 0.86 with the Hosmer–Lemeshow test)		
Age ≤ 58 years	0.0008	3.67 [1.72–7.86]
ARC detection by CG (>107.5 ml/min/1.73 m^2^)	<0.0001	4.66 [2.48–8.74]
PT	<0.0001	3.33 [1.90–5.84]
(B) Taking into account CKD-EPI equation (Goodness of fit was 1 with the Hosmer–Lemeshow test)		
Age ≤ 58 years	0.00073	2.97 1.34–6.58]
ARC detection by CKD-EPI (>108.1 ml/min/1.73 m^2^)	<0.0001	5.09 [2.74–9.48]
PT	<0.0001	3.55 [2.01–6.27]

*PT* polytrauma patients

SCr was not significant and may be a confusing factor. SAPSII was not significant in multivariate analysis and was not included in the final model. The best fit model was obtained in the model including the cut-off value of ARC detection by CKD-EPI. Goodness of fit was 1 with the Hosmer–Lemeshow test and AUC equal to 0.825. In the model including ARC detection by CG equation, the adjustment was lower (goodness of fit equal to 0.86). Taking into account these data, the logistic regression analysis showed that an age less than or equal to 58 years, an admit diagnosis of polytrauma and an eGFR above 108.1 ml/min/m^2^ as calculated by CKD-EPI appeared the only independent predictors of ARC (Table 4).

## Discussion

In ICU patients with normal serum creatinine, we found that none of the estimated GFR formulas allow accurate prediction of augmented renal clearance. However, multivariable analysis showed that 3 factors were independently correlated to ARC and useful to screen ARC: trauma patients, cut-off values of age ≤58 years, and CKD-EPI more than 108 ml/min/1.73 m².

ICU patients can exhibit important variations of their measured CrCl, despite a normal SCr with the CrCl being higher than 130 ml/min/1.73 m^2^ (ARC) in more than 33 % of the cases. In particular, younger severe trauma patients present most often with ARC compared to other groups (in our study 47 % vs. 18 %) [7]. In our sample of 360 ICU patients, the measured CrCl varied over a wide range and revealed unexpectedly low values (<60 ml/min/1.73 m^2^) in 21.4 % of the patients and very high values (>130 ml/min/1.73 m^2^) in 33.3 % of the cases. The prevalence of ARC among our patients is thus lower than in the cohort of 281 patients recently described by Udy who found an ARC in 65.1 % of cases (using the identical threshold of 130 ml/min/1.73 m^2^ to define ARC) [9]. This finding is mostly likely due to the fact that a somewhat different patient population was studied. Even though our incidence of ARC was lower, the data confirm again that in hemodynamically stable patient, a normal SCr is a poor predictor of changes in renal function [15, 31].

In 28 critically ill patients with normal SCr, Hoste demonstrated that the Cockcroft-Gault and MDRD formulas were not adequate in assessing renal function and we have previously shown similar findings in 36 burn patients [15, 31]. However, the populations of these studies were small. Our study, with 360 patients, allows a better evaluation of the large inter-individual variability presented by critically ill patients (high CrCl coefficient of variation of 51.2 %). Baptista demonstrated in 86 patients presenting ARC that both CG- and MDRD-derived values (the formula of Robert and CKD-EPI equations was not studied) significantly underestimate the measured CrCl and are insensitive in identifying ARC [16]. More recently, a study of 110 ICU patients (53 with ARC) evaluated the CKD-EPI equation and the authors showed a poor concordance with measured CrCl. It is clear that the bias and precision of these equations are significantly larger in patients with ARC [18].

In our 360 critically ill patients with normal SCr, the currently used formulas, including CKD-EPI equation, were found to be poor predictors of measured CrCl. The general increase in imprecision of estimated GFR methods at higher GFR values is well recognized [6]. Our findings, with respect to increased imprecision at measured higher creatinine clearance values, are consistent with data from the non-ICU setting. Taking into account the AUCs as well as the sensitivities and the specificities of these formulas, the CG and CKD-EPI formulas, with a cut-off, respectively, of 107.5 and 108.1 ml/min/1.73 m^2^, were found to be slightly more accurate than the other two formulas studied (Robert and sMDRD). The threshold of CG formula differs from these of Lautrette in his analysis of 32 patients admitted for acute infectious meningitis and presenting a high creatinine clearance in 47 % of the patients [1]. Our study extend on these prior works with an analysis of a large cohort of patients demonstrating again important variations of CrCl and a high prevalence of ARC that is difficult to predict based on the formulas which calculate the eGFR using the SCr.

The implications of this phenomenon primarily relate to the potential for sub-therapeutic drug levels, and treatment failures due to the correlation between CrCl and drug elimination [19]. Data provided by the Chronic Kidney Disease Epidemiology Collaboration have shown that mathematical estimates of GFR can result in up to about 20 % discordance in drug-dosing recommendations, depending on the formula used [32]. This discordance may be even higher in patients with ARC, since the population reported had significantly lower measured GFRs (75 ± 44 ml/min) compared to the patients in our study. Surprisingly, there are currently no guidelines for adjusting drug dosages for patients with an increased GFR, even though studies have clearly shown that in patients with ARC the plasma concentrations of various antibiotics (beta-lactams, vancomycin, and fluoroquinolones) were insufficient [10, 12, 14, 20, 31–37].

We acknowledge that our study has potential limitations. First, eGFRs depend on creatinine serum quantification, and the Jaffe method is prone to analytical interferences with non-creatinine compounds [24]. Our method of SCr measurement and calibration reduced these interferences [38]. Second, only direct measurement of the GFR with exogenous substances such as inulin is the gold standard for the assessment of renal function, but is not routinely performed in the intensive care units for practical reasons [39, 40]. Instead, one could measure the CrCl from a 24-h urine collection. CrCl can be affected by creatinine tubular secretion, but its impact is probably lower at higher GFRs, and we exclude patients receiving histamine-2-receptor antagonist due to its interference with tubular creatinine secretion [41]. Third, the 24-h CrCl requires steady state, and is not suited to detect rapid change in GFR [39]. Aware of these limits, we selected patients presenting SCr with less than 25 % variation between the 4th and the 10th day after admission, hemodynamically stable and without history of AKI. Furthermore, 24-h CrCl is considered imprecise in ICU practice. This method is the standard care in our unit for years, and nurse staff is well trained for 24-h urine collection. To limit bias, we mixed samples of urine bottles when diuresis was over 2 l. The quality of our 24-h urinary collection is supported by the 24-h creatinine urine excretion decreasing with CrCl [42].

A reliable way to predict the patient’s GFR is useful for the clinician and in non-ICU patients with normal GFR, a formula such as the CKD-EPI may perform reasonably well as we have recently shown to dose adjustment of vancomycin [43]. Identifying patients at risk for ARC is necessary. In our study, the multivariable analysis showed that the highest CrCls were observed more frequently in younger patients, in severe trauma patients and for cut-off values of CKD-EPI of 108.1 ml/min1/1.73 m^2^. However, the gray zone, the bias, and precision values of CKD-EPI showed the limits of these formulas, which is only a tool for screening patients with ARC. In such circumstance, the CrCl should be measured formally to accurately adjust dosage of drug eliminated by kidneys.

## Conclusion

ARC appears to be common in ICU patients especially in severe trauma patients and or in patients <58 years. The bedside measured CrCl through urine collection remains the most reliable method to detect ARC in ICU patients with normal serum creatinine levels.

This study suggests that when taking into account age and reason for admission (polytrauma and non-polytrauma), the CKD-EPI equation could allow a first screening of patients with ARC.

## Abbreviations

AKI: acute kidney injury
ARC: augmented renal clearance
AUC: area under the curve
BMI: body mass index
BSA: body surface area
CI: confidence interval
CG: cockcroft and gault
CKD-EPI: Chronic Kidney Disease Epidemiology Collaboration
CrCl: creatinine clearance
GFR: glomerular filtration rate
IBW: ideal body weight
ICU: intensive care unit
ISS: Injury Severity Score
MED: medical
NKF K/DOQI: National kidney foundation kidney/disease outcomes quality initiative
NPT: non-polytrauma
OR: odds ratio
PT: polytrauma
SAPS II: new simplified acute physiology score
SCr: serum creatinine
SD: standard deviation
sMDRD: simplified formula of the modification of diet in renal disease
SOFA: Sequential organ failure assessment
SURG: surgical
UCr: urinary creatinine
V: urinary flow rate
